# Pilot Study and Review: Physiological Differences in BDNF, a Potential Biomarker in Males and Females with Autistic Disorder

**DOI:** 10.9734/INDJ/2015/12118

**Published:** 2014-09-24

**Authors:** Eve G. Spratt, Ann-Charlotte Granholm, Laura A. Carpenter, Heather A. Boger, Carrie E. Papa, Sarah Logan, Humera Chaudhary, Sarah-Wade Boatwright, Kathleen T. Brady

**Affiliations:** 1 Department of Pediatrics, Division of Developmental and Behavioral Pediatrics, Medical University of South Carolina, Charleston, SC, United States; 2 Psychiatry and Behavioral Sciences, Medical University of South Carolina, Charleston, SC, United States; 3 Department of Neuroscience and the Center on Aging, Medical University of South Carolina, Charleston, SC, United States; 4 Department of Healthcare Leadership and Management, Medical University of South Carolina, Charleston, SC, United States; 5 School of Medicine, Medical University of South Carolina, Charleston, SC, United States; 6 Ralph H. Johnson Veterans Affairs Medical Center, Charleston, SC, United States

**Keywords:** BDNF, autism, gender, hyperactivity, brain-derived neurotropic factor

## Abstract

**Aims:**

There is a need for more biologic research in autistic disorder (AD) to determine if biomarkers exist that would be useful for correlating to symptom severity and/or clinical improvement during treatment. Given the fact that AD is 4 times more common in males than females, gender differences in physiological biomarkers may be present. One potential biomarker that has begun to be studied is brain-derived neurotropic factor (BDNF), a peptide involved in the regulation of neuronal cell survival, differentiation, and plasticity, and possessing an ability to influence neurotransmitter systems by modulating gene expression. This pilot study examined whether serum BDNF differed according to gender in children with AD and whether differences were associated with a behavioral phenotype or severity of illness.

**Study Design:**

Data for this investigation were collected during the participants’ baseline visit of an intervention study. Participants were males (n=29) and females (n=7), aged 5 to 12 years diagnosed with AD. Baseline serum BDNF concentration was determined for comparison to clinical ratings using an autism severity measure and the Pervasive Developmental Disorder-Behavior Inventory (PDD-BI).

**Results:**

BDNF serum concentrations were higher in females (p<0.049). The baseline BDNF value corresponded significantly to hyperactivity in females (p<0.0002) but not in males. BDNF did not correlate with severity of disease in either gender.

**Conclusion:**

Although this is a small study, a better understanding of the central role of BDNF may provide insight into the pathophysiology of the disease and elucidate why gender differences exist in prevalence and behavioral phenotype of AD.

## 1. INTRODUCTION

Autistic Disorder (AD) is a developmental disorder characterized by deficits in language and social interaction, as well as the presence of atypical or repetitive behaviors. The etiology of AD remains unknown; however, a clear gender bias exists as the disorder is 4 times more common in males. In 2008, approximately 1 in 54 males and 1 in 252 females were found to have an autistic disorder [1]. There is a paucity of research examining gender differences in AD and the existence of biological pathways that influence severity and gender differences are poorly understood. An improved understanding of neurobiological factors may lead to more effective therapeutic interventions and an improved understanding of the etiology and pathophysiology of the disorder.

Neurotropic factors are important regulators of neuronal growth, cell differentiation, and cell survival during early brain development. Brain derived neurotropic factor (BDNF) plays a critical role in synaptogenesis and synaptic plasticity [2]. This small protein is found throughout the brain, CNS and in peripheral blood. BDNF levels in the blood appear to reflect BDNF levels in the brain [3]. Because of its wide range of actions and presence in multiple brain regions and neuronal cell types, levels of BDNF have been studied in a multitude of mental illnesses including mood disorders, psychosis, eating disorders, and autism. BDNF plays a role in the survival and differentiation of dopaminergic neurons in the developing brain and plays a key role in the plasticity of the brain [4–6]. BDNF is trophic for serotonergic neurons, a finding that may be associated with abnormal serotonin levels found in autism [7]. This pilot project was developed to better understand if gender differences in BDNF levels in children with AD might exist and to explore the relationship between serum BDNF levels and specific psychological and cognitive markers as well as severity of the disease.

Reports of altered BDNF in autism have most commonly found elevated values from normal. In one study, higher BDNF levels were found in neonatal blood from children with AD [8]. Other studies have shown higher concentrations of serum BDNF in older children (ages 3 to 27) with autistic disorders [9] as well as higher BDNF levels in the basal forebrain in adults with AD compared to healthy controls [10]. Higher levels of serum BDNF are consistent with the observations of an increase of the number of neurons in the prefrontal cortex of autistic disorder patients [11,12]. One possible explanation of differences from controls may be a role of age related delays in BDNF levels in comparison to youth with normal development. A recent study found that BDNF was elevated in 29 patients with AD as compared to age and gender matched controls [13]. This study also found elevated levels of the pro inflammatory cytokines including interleukin-1, interleukin-6, interleukin 12, interleukin-23, and tumor necrosis factor, suggesting an influence of immunoexcitotoxic modulators in the pathophysiology of AD. The dysregulation of cytokine and neurotrophin levels could play a contributory role in the etiopathogenesis of AD [13]. However, some studies of BDNF levels in AD have different findings. Katoh-Semba, Wakako [14] found lower BDNF levels in the serum of subjects with autism as compared to age matched healthy control subjects. Das [15] reported that in children with autism, BDNF plasma levels were significantly lower compared to healthy controls and it was hypothesized that apoptosis may play a role in the BDNF-Akt-Bc12 anti-apoptotic signaling pathway as a mechanism contributing to the pathology of AD. These studies above did not report whether BDNF differed by gender, severity of illness or specific behaviors.

BDNF has been found to be lower in subjects with other brain based disorders and mental illness such as major depressive disorder and bipolar disorder. BDNF has been found to be decreased in lymphocytes and platelets of patients with pediatric bipolar disorder compared to controls [16]. Karege et al. [17] found serum BDNF levels to be significantly lower in subjects with major depressive disorder compared with normal controls. Shimizu et al. [18] determined BDNF serum levels to be significantly lower in subjects with major depressive disorder who were not taking anti-depressant medications compared with depressed subjects taking antidepressants or normal control subjects. Additionally, Pandey et al. [16] determined non-medicated pediatric patients diagnosed with bipolar disorder to have significantly decreased BDNF mRNA levels in the lymphocytes in comparison with healthy control subjects. In the same study, BDNF protein levels in platelets were significantly lower compared to the healthy control subjects. In subjects with bipolar disorder, lower serum BDNF levels were found when the subjects were in both the manic or depressive state compared with normal controls [19].

However, appropriate medications and exercise have been shown to increase BDNF levels and this appears to be associated with improved brain health. In one study, pediatric bipolar patients treated with mood stabilizing drugs demonstrated a positive change with increased levels of BDNF mRNA in the lymphocytes similar to those of normal control subjects [16]. In individuals without brain based disorders, research has identified that exercise induces a rapid induction of BDNF mRNA and protein in individuals [20–22]. This is an especially interesting finding given that exercise has been shown to be an effective strategy to improve cognitive health and the plasticity of the nervous system [21,23]. BDNF is known to support and stimulate neuronal survival and growth as it is a key mediator of synaptic efficacy, neuronal connectivity, and synaptic plasticity [20,24–26]. An improved understanding of the role BDNF plays in the brains of individuals with AD may provide evidence for the pathophysiology of the disease and an improved understanding of factors that increase BDNF may lead to improved mental health and a reduction of symptom severity.

## 2. MATERIALS AND METHODS

Participants were 36 children (29M: 7F; mean=9.0years) with a history of Diagnostic and Statistical Manual of Mental Disorders-IV (DSM-IV) AD, who met criteria for AD on the Autism Diagnostic Observation Schedule (ADOS). Children were recruited for a fatty acid intervention study (PI: author LC) from the Division of Genetics and Developmental Pediatrics at the Medical University of South Carolina, from the South Carolina Autism Society, and the South Carolina Department of Disabilities and Special Needs through direct clinic approach, direct mailings, and newsletter advertisements. Participants enrolled were healthy children ages 5–12 who had been diagnosed with Autism through administration of the Autism Diagnostic Observation Schedule (ADOS) [27]. Baseline labs included BDNF measures for this exploratory study. The mean age of 7 females was 9.14 years and the mean age of 29 males was 8.90 years. The ethnic distribution included 67% Caucasian, 25% African-American, and 8% other. All were participating in a treatment study with fatty acids. Data for the current investigation were collected during the participants’ baseline visit, prior to any research intervention. The study data included baseline serum BDNF levels, an autism severity measure, and the Pervasive Developmental Disorder-Behavior Inventory (PDD-BI). Severity of Illness indicates the severity of Autistic Disorder as rated on the Clinical Global Impression Scale (CGI). Blood was collected according to standard procedures in the South Carolina Translational Clinical Research Center by using experienced nursing staff using a small gauge needle, immediately put on ice after blood collection, centrifuged, and serum samples were frozen at −20°C until use.

### 2.1 BDNF ELISAs

BDNF protein levels were assessed in serum samples using a commercially available assay kit from R&D (R&D Systems, Minneapolis, MN). Flat-bottom plates were coated with the BDNF capture antibody. Samples were centrifuged for 20 min and then incubated for 2 hours. The captured BDNF was bound by a second specific antibody, which was detected using a species-specific antibody conjugated to horseradish peroxidase as a tertiary reactant. All unbound conjugates were removed by subsequent wash steps. After an incubation period with chromagenic substrate, color change was measured in an ELISA plate reader at 450 nm. Using this method, BDNF can be quantified in the range of 7.8–500 pg/ml with a cross-reactivity of <2–3%.

Statview was used for statistical tests conducted by the second author. A basic Pearson correlation matrix was applied to examine correlations between different outcome measurements. For direct comparisons between two or more factors, ANOVA assessment with Bonferroni post hoc comparison was applied.

## 3. RESULTS AND DISCUSSION

**Fig. 1:**
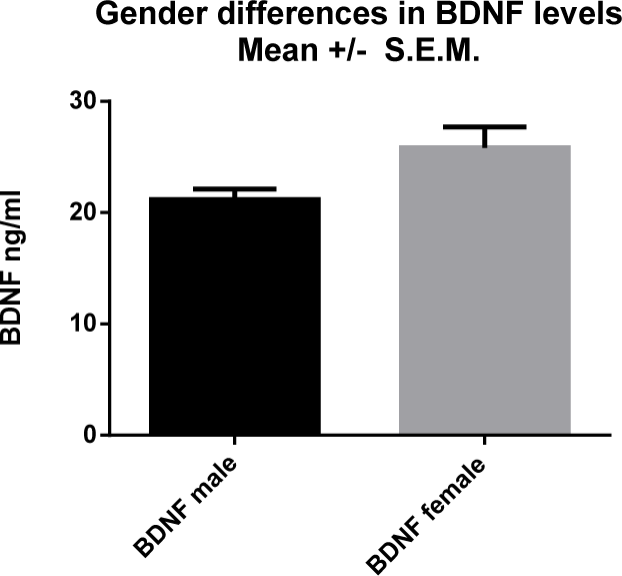
Gender Differences in BDNF Levels (ng/mL) A) BDNF levels corresponded significantly to hyperactivity in females (p<0.0002) but not in males (p=0.29)

Analysis revealed significantly higher serum levels of BDNF in samples from females compared to males (p<0.049) with no significant difference among ethnic groups (Tables 1 and 2; Fig. 1). BDNF levels correlated significantly to the hyperactivity subscale on thePDD-BI for females only (Table 3). Females showed a strong correlation between BDNF levels and hyperactivity (p=0.0002; n=7), while males did not show any significant correlation for these two markers (p=0.29; n=29). The hyperactivity subscale did not correlate significantly with severity of illness on the CDI for males (p=0.195) or females (p=0.336; Table 3). Neither males nor females had significant correlations between AD severity and BDNF levels. No other measures correlated with BDNF.

The *F* test statistic had the value of 4.27. Using an*α* of 0.05, the critical value was 4.21 (*F* 0.05; 1, 27). Since the test statistic is larger than the critical value, we reject the null hypothesis of equal means between the gender groups and conclude that there is a statistically significant difference among the means (p=.049) (Table 1).

The *F* test statistic had the value of 4.27. Using an*α* of 0.05, the critical value was 4.21 (F 0.05; 1, 27). Since the test statistic is larger than the critical value, we reject the null hypothesis of equal means for Corrected BDNF (ng/mL) between the gender groups and conclude that there is a statistically significant difference in means between boys and girls (p=.049) (Table 1).

In this small sample baseline BDNF did not predict a change in boys or girl’s hyperactivity or overall Autism rating scales.

## 4. CONCLUSION

In this small exploratory study we assessed the role of gender, AD symptom characteristics, and severity in relation to BDNF levels in children with autistic disorder. Several studies have indicated that BDNF plays a significant role in the neurobiology of autism [15,28,29]. Abnormalities in BDNF levels in other brain based mental health disorders have been noted. This was an exploratory study with a small number of subjects yet findings revealed significant gender difference results. This indicates a need for replication and for contributing influences to be explored and better understood. Females had significantly higher levels of BDNF than males and BDNF levels were significantly correlated with the hyperactivity subscale on the PDD-BI for females.

The correlation between high psychomotor activity level and elevated BDNF serum levels found in this pilot study may have relevance for hyperactivity related to autism and warrants further investigations. This is especially interesting given research that has found BDNF levels to be higher for individuals that actively exercise [23].

## 5. LIMITATIONS

There are a few limitations in our study. The sample size is small with a limited representation of females. The fact that we found gender differences and specifically, a relationship between BDNF and hyperactivity characteristics in females, suggests that gender differences related to BDNF as well as the relationship of BDNF to hyperactivity and overall activity level may be real but requires replication. Although the presence of depression in our population would limit the interpretation of an association with AD, this population was young and depression was not a diagnosed co-morbidity in any participants. In addition, it is not firmly established that serum levels of BDNF adequately reflect brain levels, but previous research has found brain levels to be consistent with peripheral levels [16]. The previous studies in other psychiatric disorders such as major depression and bipolar illness have strongly suggested BDNF serum levels may be used as a biomarker for onset or progression of symptoms or improvement after treatment. BDNF may have the potential to be a marker of symptom improvement after behavioral and medication treatments in AD. Additional study is needed about the usefulness of BDNF as a biomarker in conjunction with monitoring of exercise, use of medications and cytokine activity in conjunction with gender differences and changes in symptoms and severity of illness. We realize the need for cautious interpretation of our findings given the small number of subjects. Future research plans to replicate these findings will include a healthy age matched control group without AD to use for comparison as this may uncover additional clues to related to the pathophysiology of Autism and the gender differences that exist.

## Figures and Tables

**Table 1:** ANOVA table for corrected BDNF (ng/mL)

	DF	Sum of squares	Mean square	F-value	P-value	Lambda	Power
Gender	1	215.675	215.675	4.266	.049	4.27	.50
Residual	27	1365.056	50.558				

**Table 2:** Corrected BDNF (ng/mL) and hyperactivity

	Correlation	Count	Z-value	P-value	95% lower	95% upper
Males only	−.409	9	−1.063	.29	−.844	.351
Females only	.951	7	3.677	.0002	.695	.993

Correlation Coefficient; Hypothesized Correlation = 0; Row exclusion: Biologic + Behavioral n=36

B) There were no gender differences in terms of severity of illness or hyperactivity. C) Hyperactivity did not correlate with severity of disease

**Table 3:** Hyperactivity and severity of illness**

	Correlation	Count	Z-value	P-value	95% lower	95% upper
Males only	.485	9	1.30	.195	−.265	.87
Females only	.447	7	.96	.336	−.461	.90

Correlation Coefficient; Hypothesized Correlation = 0; Row exclusion: Biologic + Behavioral n=36

** Severity of Illness indicates the severity of Autistic Disorder as rated on the Clinical Global Impression Scale (CGI)

## References

[1] Center for Disease Control (2008). Prevalence of autism spectrum disorders - Autism and Developmental Disabilities Monitoring Network (ADDM), 14 sites. United States.

[2] Lang UE, Jockers-Scherubl MC, Hellweg R (2004). State of the art of the neurotrophin hypothesis in psychiatric disorders: Implications and limitations. J Neural Transm.

[3] Garcia KL, Yu G, Nicolini C, Michalski B, Garzon DJ, Chiu VS (2012). Altered balance of proteolytic isoforms of pro-brain-derived neurotrophic factor in autism. J Neuropathol Exp Neurol.

[4] Binder DK, Scharfman HE (2004). Brain-derived neurotrophic factor. Growth Factors.

[5] Hyman C, Hofer M, Barde YA, Juhasz M, Yancopoulos GD, Squinto SP (1991). BDNF is a neurotrophic factor for dopaminergic neurons of the substantia nigra. Nature.

[6] Teixeira AL, Barbosa IG, Diniz BS, Kummer A (2010). Circulating levels of brain-derived neurotrophic factor: Correlation with mood, cognition and motor function. Biomark Med.

[7] Al-Ayadhi LY (2012). Relationship between Sonic hedgehog protein, brain-derived neurotrophic factor and oxidative stress in autism spectrum disorders. Neurochem Res.

[8] Nelson KB, Grether JK, Croen LA, Dambrosia JM, Dickens BF, Jelliffe LL (2001). Neuropeptides and neurotrophins in neonatal blood of children with autism or mental retardation. Ann Neurol.

[9] Miyazaki K, Narita N, Sakuta R, Miyahara T, Naruse H, Okado N (2004). Serum neurotrophin concentrations in autism and mental retardation: A pilot study. Brain Dev.

[10] Perry EK, Lee ML, Martin-Ruiz CM, Court JA, Volsen SG, Merrit J (2001). Cholinergic activity in autism: Abnormalities in the cerebral cortex and basal forebrain. Am J Psychiatry.

[11] Courchesne E, Mouton PR, Calhoun ME, Semendeferi K, Ahrens-Barbeau C, Hallet MJ (2011). Neuron number and size in prefrontal cortex of children with autism. JAMA.

[12] Lainhart JE, Lange N (2011). Increased neuron number and head size in autism. JAMA.

[13] Ricci S, Businaro R, Ippoliti F, Lo Vasco VR, Massoni F, Onofri E (2013). Altered cytokine and BDNF levels in autism spectrum disorder. Neurotox Res.

[14] Katoh-Semba R, Wakako R, Komori T, Shigemi H, Miyazaki N, Ito H (2007). Age-related changes in BDNF protein levels in human serum: Differences between autism cases and normal controls. International Journal of Developmental Neuroscience.

[15] Das UN (2013). Nutritional factors in the pathobiology of autism. Nutrition.

[16] Pandey GN, Rizavi HS, Dwivedi Y, Pavuluri MN (2008). Brain-derived neurotrophic factor gene expression in pediatric bipolar disorder: Effects of treatment and clinical response. J Am Acad Child Adolesc Psychiatry.

[17] Karege F, Perret G, Bondolfi G, Schwald M, Bertschy G, Aubry JM (2002). Decreased serum brain-derived neurotrophic factor levels in major depressed patients. Psychiatry Res.

[18] Shimizu E, Hashimoto K, Okamura N, Koike K, Komatsu N, Kumakiri C (2003). Alterations of serum levels of brain-derived neurotrophic factor (BDNF) in depressed patients with or without antidepressants. Biol Psychiatry.

[19] Cunha AB, Frey BN, Andreazza AC, Goi JD, Rosa AR, Goncalves CA (2006). Serum brain-derived neurotrophic factor is decreased in bipolar disorder during depressive and manic episodes. Neurosci Lett.

[20] Intlekofer KA, Cotman CW (2013). Exercise counteracts declining hippocampal function in aging and Alzheimer’s disease. Neurobiol Dis.

[21] Cotman CW, Berchtold NC (2002). Exercise: A behavioral intervention to enhance brain health and plasticity. Trends Neurosci.

[22] Neeper SA, Gomez-Pinilla F, Choi J, Cotman C (1995). Exercise and brain neurotrophins. Nature.

[23] Gomez-Pinilla F, Hillman C (2013). The influence of exercise on cognitive abilities. Compr Physiol.

[24] Lu B, Chow A (1999). Neurotrophins and hippocampal synaptic transmission and plasticity. J Neurosci Res.

[25] McAllister AK, Katz LC, Lo DC (1999). Neurotrophins and synaptic plasticity. Annu Rev Neurosci.

[26] Schinder AF, Poo M (2000). The neurotrophin hypothesis for synaptic plasticity. Trends Neurosci.

[27] Lord C, Rutter M, DiLavore P, Risi S (2003). Autism Disgnostic Observation Schedule.

[28] Chapleau CA, Larimore JL, Theibert A, Pozzo-Miller L (2009). Modulation of dendritic spine development and plasticity by BDNF and vesicular trafficking: Fundamental roles in neurodevelopmental disorders associated with mental retardation and autism. J Neurodev Disord.

[29] Raznahan A, Toro R, Proitsi P, Powell J, Paus T, FB P (2009). A functional polymorphism of the brain derived neurotrophic factor gene and cortical anatomy in autism spectrum disorder. J Neurodev Disord.

